# Effect of Hypoxic Injury in Mood Disorder

**DOI:** 10.1155/2017/6986983

**Published:** 2017-06-22

**Authors:** Fenglian Zhao, Junling Yang, Ranji Cui

**Affiliations:** Jilin Provincial Key Laboratory on Molecular and Chemical Genetics, The Second Hospital of Jilin University, Changchun, Jilin 130041, China

## Abstract

Hypoxemia is a common complication of the diseases associated with the central nervous system, and neurons are highly sensitive to the availability of oxygen. Neuroplasticity is an important property of the neural system controlling breathing, memory, and cognitive ability. However, the underlying mechanism has not yet been clearly elucidated. In recent years, several pieces of evidence have highlighted the effect of hypoxic injury on neuronal plasticity in the pathogenesis and treatment of mood disorder. Therefore, the present study reviewed the relevant articles regarding hypoxic injury and neuronal plasticity and discussed the pathological changes and physiological functions of neurons in hypoxemia in order to provide a translational perspective to the relevance of hypoxic injury and mood disorder.

## 1. Introduction

The ability of the brain to absorb internal and external information, learn new skills, and form new memories is dependent on neural functions. Neurons are the fundamental structural and functional units in the neural information network. The neural plasticity might be an ability of the nervous system to adapt in response to intrinsic and extrinsic stimuli [1]. A large number of studies demonstrate that neural circuits, synaptic connections, morphology, and the biochemical components (including nucleic acids, enzymes, and neurotransmitters) of a single neuron may exhibit a certain degree of plasticity. This phenomenon explicates why dysregulation or disruption of neural plasticity may be associated with neurodegenerative and neuropsychiatric disorders, such as mood disorders. Mood disorders are known as mental disorders characterized by periodic elevation of the mood, which might sometimes alternate with the periodic depression. The pathogenesis of mood disorders is yet uncertain, and the role of neural plasticity in mood disorder has been widely evaluated.

Recently, a large number of factors have been identified that can affect neural plasticity; the correlation between hypoxic injury and neural plasticity has been under intensive focus. In this article, we review some of the recent studies on the relationship of hypoxic injury and neural plasticity in mood disorders and explore the mechanism underlying the hypoxic damage leading to mood disorders.

## 2. The Main Mechanism of Hypoxic Injury

Hypoxemia refers to insufficient oxygen in the circulating blood. However, the general term hypoxia indicates an abnormally low concentration of oxygen in any tissue, organ, or the body as a whole. Hypoxia has been implicated in a large number of pathologies, including head trauma, stroke, neurodegenerative diseases, obstructive sleep apnea (OSA), chronic obstructive pulmonary disease (COPD), and interstitial lung disease (ILD), which are related to the central nervous system (CNS) and respiratory system. Thus, any factors that affect the volume or rate of oxygen reaching the lungs or any causes that lessen the transfer of oxygen from the lungs to the blood might result in hypoxemia. The brain is highly sensitive to the oxygen concentration in the artery [2]. Any disease that affects the airflow and blood perfusion could cause decreased oxygen supply to the brain [3] that might alter the neuronal function, leading to cell injury and death. Spatial memory and learning deficits caused by long-term intermittent hypoxia (IH) are accompanied by an increase in oxidative stress in the brain areas, such as the hippocampus, involved in cognition and memory [4]. Hypoxia causes the enhanced anaerobic glycolysis in cells [5, 6], which led to aberrant oxidative phosphorylation and energy supplement in cells. In addition, patients with COPD and obstructive sleep apnea are often accompanied by systemic inflammation [7, 8], which might aggravate the neuronal damage. Hypoxia-induced neurodegeneration and apoptosis may also play a vital role in mood disorder. Protocatechuic acid (PCA) was noted to alleviate the oxidative stress, apoptosis, and glial proliferation; moreover, it also decreased the level of IL-1*β* in the brain following chronic intermittent hypoxia, further enhancing the learning and memory ability [9].

In addition, ischemia is commonly a restriction in blood supply to tissues, which result in the deficiency of glucose and oxygen needed for cellular metabolism. Ischemia includes insufficiency of oxygen, decreased availability of nutrients, and inadequate discharge of metabolic rubbish. Both hypoxic injury and ischemic damage effectuate in coordination during the pathogenic process of different diseases, such as stroke.

### 2.1. Oxidative Stress

Oxidative stress is caused by increased production of both reactive oxygen species (ROS) and reactive nitrogen species (RNS) or decreased antioxidant ability and the capacity of elimination of free radicals. When the oxygen supply is not sufficient, the electron transport chain is impeded, and oxygen radicals and peroxynitrite can be produced, including hydrogen peroxide, superoxide, perhydroxyl, and hydroxyl radicals. This process can be aggregated by Ca^2+^ accumulation in the mitochondria that results in mitochondrial dysfunction which, in turn, increases the production of reactive oxygen radicals. All these ROS/RNS can alter the structure of DNA by direct interaction and lead to cell injury and apoptosis [10]. Hypoxia also induces the cells to undergo oxidative stress from the uncontrolled generation of ROS in the mitochondrion that might lead to cell death in the tissue [6, 11, 12]. The hypoxia and ischemia-reperfusion injury are severe in some brain injury patients; the abundant production of ROS/RNS and inadequate supply of antioxidants is primarily responsible for the subsequent brain pathology, as the balance between the oxidative and antioxidative systems is disrupted in the brain [13, 14]. However, the chronic intermittent hypoxia (CIH) is shown to occur in the respiratory diseases, causing an increase in ROS/RNS and the overall oxidative stress [13, 14]. Furthermore, oxidative stress is associated with the development of CNS diseases involving cognitive function and memory processes [15–17], such as hippocampal synaptic plasticity. Hypoxia-induced oxidative stress also contributes to neurodegeneration and apoptosis [18], as well as spatial memory impairment [11].

### 2.2. Systemic Inflammation

Systemic inflammation is crucial for the mechanism of hypoxic brain injury [19, 20]. The acute and chronic inflammation event that occurs during and following hypoxia may be involved in secondary neuronal injury processes [10]. The process of inflammation is characterized by the infiltration of inflammatory cells, production of inflammatory mediators, and activation of resident cells. The activation and infiltration of inflammatory cells into a functional brain area is the interactive effect of a series of adhesion molecules, chemokines, and cytokines. The cytokines involved in the process of hypoxic injury include tumor necrosis factor alpha (TNF-*α*), transforming growth factor beta (TGF-*β*), interleukin-6 (IL-6), and other factors. Various cytokines and chemokines have been produced and released during the inflammatory process; furthermore, neutrophils, macrophages, T cells, and other cells, have been activated, which could aggravate the secondary injury of the tissue [10]. The prognosis of acute and prolonged inflammatory process in the brain may be different depending on the ether interaction between the inflammatory cells and mediators or the compensatory capacity of the body.

Overall, the chronic systemic inflammation may result in cognitive function impairment by the following aspects: (1) persistent activation of microglia induces neuronal injury [21]; (2) inflammation leads to the morphological changes in neuronal dendritic spines in susceptible regions in the brain [22]; and (3) inflammation downregulates the expression of the genes associated with growth factors that are involved in cognitive and memory ability [22].

### 2.3. Apoptosis

Apoptosis is a genetically programmed cell death occurring at specific developing stages in plants and animals; it is an active death-related process of the cells and is controlled by specific genes [23, 24]. The biochemical events lead to characteristic morphological changes and death in the cells. These characteristic changes include cell blebbing, cell shrinkage, nuclear chromatin condensation and fragmentation, chromosomal DNA fragmentation, and mRNA decay [10]. In normal physiological conditions, apoptosis is a homeostatic mechanism maintaining the cells in different tissues. Apoptosis also occurs as a protective response in the case of cell damage in abnormal pathological conditions. Apoptosis is an energy-dependent coordinated process that involves the activation of a series of cysteine proteases and a complex cascade of events that link the initiating stimuli to the final cell death [23]. In some cells, radiation treatment can lead to apoptosis through a p53-dependent pathway-mediated DNA damage, as well as other cellular apoptosis reactions induced by expressing Fas or TNF receptors [23]. A recent study suggests that delayed hyperbaric oxygen can enhance neurogenesis and improve neuronal function by ROS/HIF-1*α*/*β*-catenin pathway [25].

### 2.4. Endoplasmic Reticulum and Excitotoxicity

The endoplasmic reticulum (ER) is a locus where proteins are modified, folded, and calcium-modulated. Reportedly, ER can prevent oxidative stress and aberrant Ca^2+^ regulation from killing the neurons in vivo and in vitro [26–28]. ER stress is initiated in the early stage of hypoxic-ischemia, and the activation of ER stress response is a grave consequence of enhanced oxidative stress, which could result in a series of cellular malfunctions as well as neuronal apoptosis [29, 30]. ER stress can cooperate with autophagy in the process of neurodegeneration with the morphological changes in the ER structure [31]. Regular exposure to the intermittent hypoxic environment can cause dilation and distortion of ER in the hippocampus of mice that is accompanied by excessive expression of Grp78 [32], caspase-12, and CHOP; caspase-12 and CHOP are the two mediators associated with ER-induced apoptosis [33, 34].

CIH exposures lead to upregulation of the unfolded protein response (UPR) in the brain prefrontal cortex and hippocampus by enhanced phosphorylation of PKR-like ER kinase, which indicates that a specific ERS inhibitor, salubrinal, protects the neurons against CIH-induced injury [35]. The short-lasting activation of UPR can prevent its further accumulation by inhibiting the synthesis of protein, and the sustained activation of UPR might be associated with the neurodegeneration in hypoxia-ischemia [36].

Thus, the above findings suggest that ER is a valuable biomarker of the severity of hypoxia-ischemia injury.

## 3. Hypoxic Injury and Neuronal Plasticity

The brain is highly sensitive to the change in the arterial concentration of oxygen [2]; it ineluctably sustains the hypoxic stress. Decreased oxygen content might lead to neuronal injury in the brains of COPD patients, which is demonstrated by clinical symptoms such as mood disorders and neuropsychological deficits [3]. Furthermore, the systemic inflammation is accompanied by a hypoxic injury that may exacerbate the neuronal injury. A large number of patients with intermittent hypoxia manifest a series of symptoms related to the injury to the nervous system, which is exhibited as a deficiency in memory, learning, and decision-making ability [37]. In addition, the hypoxic damage in the brains of other patients is manifested as depression, anxiety, physical disabilities [7], and neuropsychological deficits [2, 38–41]. Compared to the controls, patients with COPD displayed a poor performance in the figure memory test, mini-mental state examination, and visual reproduction [3]. Some studies demonstrate that hypoxia is associated with the changes in the brain structure, including volume atrophy and a decrease in the gray matter in the amygdala, hippocampus, anterior cingulate cortex, prefrontal cortex, and other regions [42, 43]. Different degrees of IH-induced irreversible damage to the neural cell ultrastructure and loss of nerve cells, which could cause various degrees of nervous system disorders. The neuronal plasticity plays a critical role in brain function. The hypoxic injury affects the neuronal plasticity in the brain through different mechanisms, thereby affecting the brain in response to intrinsic and extrinsic stimuli. Moreover, several regions of the brain, such as the hippocampus, frontal cortex, amygdala, insula, anterior cingulate, fornix, mammillary bodies, and cerebellum, are impaired during and after sleep-disordered breathing, and all these structures are altered in depression patients [44]. The different kinds of changes of neural plasticity induced by hypoxic-ischemic injury in various brain regions are shown in Table 1.

### 3.1. The Hypoxic Injury in the Hippocampus

In the brain, the hippocampus has an affinity with learning and memory, and the hippocampal synapse is a model system to study the mechanisms of learning and memory [45]. The hippocampal synaptic plasticity is considered as important for the formation of hippocampus-dependent explicit memory [46]. A previous study on quantum dots proved that nanoparticle impaired the synaptic transmission and plasticity in the hippocampus dentate gyrus area of rats via oxidative stress [47]. The long-term synaptic plasticity is an essential property of the nervous system and a critical mechanism for memory and learning, which can bidirectionally modify the synaptic strength—either depressing (long-term depression (LTD)) or enhancing (long-term potentiation (LTP)) [48]. Some studies show that NO affects the hippocampal synaptic plasticity, including LTD and LTP, and consequently learning and memory [49–54]. Also, NO serves as a retrograde messenger and acts directly on the presynaptic neurons to produce LTP [48, 55–57] and then suppresses the synaptic plasticity [58]. Other studies have shown that some drugs could protect the hippocampal neurons by inhibiting the NOS activity and expression in the hippocampus [58–60]. The ER stress induced by chronic IH resulted in apoptosis and fine structural changes in the synapses in the hippocampal CA1 region [61], which might inhibit the transfer of Schaffer collaterals from CA3 to CA1 neurons [62]. Hypoxia and inflammation act in synergy to trigger the long-term synaptic depression (LTD), which might contribute towards synaptic disruptions and memory impairments in neuroinflammation-related brain disorders [63].

MAPK phosphorylation is enhanced following IH. The overstimulation of MAPK signaling pathways by IH induces the abnormal expression of Bax and Bcl-2, resulting in a severe loss of hippocampal neurons. This phenomenon was correlated to oxidative stress, inducing the upregulation of malondialdehyde (MDA) and the downregulation of superoxide dismutase (SOD) [64]. Accumulating evidence demonstrate that the production of ROS is closely related to protein folding [65, 66], and the accumulation of misfolded or unfolded protein could cause increased ROS production [29]. The present study suggests that the aberrant energy metabolism might be an alternative mechanism underlying depression.

In addition, the volumetric changes of the hippocampus are also critical to mood regulation. Reportedly, the cognitive disabilities in hypoxia-ischemic encephalopathy patients are also accompanied by hippocampal atrophy; the white matter density is decreased in the frontal gyrus [67, 68]. In the event of hypoxia induced by central hypoventilation syndrome (CHS), the hippocampus shows a reduced volume [69]. Smaller hippocampal volumes have also been reported in schizophrenic individuals who suffered pre- and perinatal hypoxia [70]. Additionally, some studies postulate that hippocampal apoptosis is a causative factor in hippocampal volumetric changes [71].

### 3.2. The Hypoxic Injury in the Prefrontal Cortex

The prefrontal cortex (PFC) is a crucial nerve center of thinking and behavior regulation in the brain [72] and is divided into two subregions: ventromedial prefrontal cortex (vmPFC) for regulating the affection and dorsolateral sectors (dlPFC) for mediating the cognitive functions [73, 74]. The interactions within the neuronal networks centered on the hippocampus and PFC are closely related to information transfer and storage [75–77]. The PFC receives strong monosynaptic projections from the intermediate and ventral hippocampus [78, 79]. The hypoxia-ischemic injury causes the disruption of interactions within the prefrontal-hippocampal networks [80], which could affect the thinking and behavior. Several studies reported that the frontal lobe dysfunction led to various degrees of cognitive impairments such as inattention, hyperactivity, impulsiveness, and changes in the personality [81].

PFC is a region contributing towards the behavioral regulation and neuroendocrine responses to stress and can be injured by excessive exposure to a stimuli-induced release of inflammatory mediators and steroid molecules [82–84]. A previous study reported that animals exposed to IH presented impaired executive function associated with dopaminergic disturbance and tissue atrophy in the PFC [85]. The increased density of GABAergic neurons in the PFC during and after IH disrupts the balance between excitation and inhibition which, in turn, impairs the network activity within PFC [80]. Since the thalamic burst spikes are efficient in exciting the postsynaptic neurons [86], the activation of T-type Ca^2+^ channels in the MD might specifically stimulate the PFC neurons and then lead to frontal lobe-specific seizures [81]. In the mouse model, PFC damage induced by hypoxic conditions including ghost cells, increased the neuronal death and upregulated the expression of molecules such as VEGF, Glut1, Hif-1, and lactate dehydrogenase A, which might cause a local excitability in PFC neurons [81, 87]. In addition, NADPH oxidase-2 induced the oxidative stress that might contribute to the developmental loss of parvalbumin- (PV-) positive cells (PV-cells) in the PFC and progression of psychiatric anxiety in rat models [72, 88]. Moreover, the loss of PV-cells in the PFC and the IH-induced psychiatric anxiety can be alleviated by inhibiting the NADPH oxidase-2-induced oxidative stress [88].

Acetylcholine is an indispensable element for memory and learning [89]. IH could induce spatial learning deficits, and the expression of choline acetyltransferase in the basal forebrain decreased in rats [90, 91]. Muscarinic cholinergic receptors are coupled to G proteins [92], and M2-muscarinic receptor regulates the release of acetylcholine in PFC in mouse [93]. G proteins constitute a functionally complex network that enhances the transmembrane signaling. A few changes in the activation of G proteins can dramatically cause distal signal transduction cascades. Hypoxia enhanced the cholinergic activation and mu-opioid activation of G proteins in the PFC, thereby increasing the acetylcholine release of PFC neurons; thus, the functions of PFC is altered [94].

### 3.3. The Hypoxic Injury in the Amygdala

The amygdala is closely associated with emotion, learning, attention, and memory, especially its role in the correlation between negative emotion and learning ability as well as memory [95]. The disturbances to the amygdala constitute the primary features of bipolar disorder [96]. The various changes in the morphology and function of amygdala might be related to mood disorders such as depression [97, 98]. The hypoxic injury arising from diverse factors might cause serious changes in the amygdala and has been demonstrated in several animal models. Carty et al. found a significant decrease in the cell size and axonal degeneration of corticotropin-releasing factor-positive neurons in the amygdala of the rat after undergoing neonatal hypoxia-ischemia [99]. The gray matter in the amygdala is reduced in children who suffered from neonatal hypoxia-ischemia [100], and the volume of the left amygdala is reduced in patients with COPD [3]. The number of corticotropin-releasing factor- (CRF-) and neuropeptide-Y- (NPY-) positive neurons were found to be decreased distinctly in the amygdala after postnatal day 3 hypoxia-ischemia [99], and c-fos protein expression increased in the nc. accumbens and the anterior amygdaloid area in the rat brain exposed to hypoxic injury [101]. These long-term changes in the amygdala may be functionally associated with the specific behavioral disorders including bipolar disorder [102].

However, some researchers found that perinatal hypoxia does not change the susceptibility to amygdaloid-induced seizures in the adult rabbits [103].

### 3.4. The Hypoxic Injury in Other Structures of the Brain

Heart failure (HF), OSA, and congenital central hypoventilation syndrome (CCHS) occur as a result of insular and cingulate cortex injury [62]. Damage in those regions apparently contributes towards inhibiting or enhancing the sensation. The extent of injury in the insula shows the loss of tissue in HF, increased mean diffusivity in OSA, and axial and radial diffusivity changes in CCHS as well as in anterior cingulate [62]. Other studies have shown that the cortical thinning and white matter loss also occurs in the brain after the repeated chronic exposure to hypoxia and hypercapnia in CHS [104, 105]. Based on the fMRI study, significant differences were observed in the magnitude and timing of responses in specific regions of the brain between groups induced by hypoxia; these regions included the cerebellar cortex, deep nuclei, and posterior thalamic structure, as well as the amygdala and hippocampus. These finds emphasize the important roles of posterior thalamus, midbrain, and cerebellum in normal hypoxic conditions [106].

### 3.5. The Related Signaling Pathway in Hypoxia/Hypoxic-Ischemia Injury

Several pathways, regulators, and effectors participate in the pathological process of the secondary injury of brain hypoxia/hypoxia-ischemia reperfusion. The damage to blood-brain barrier (BBB) also plays a critical role in the initiation of the reoxygenation/reperfusion injury and development.

Hypoxia/reoxygenation (H/R) stress can induce the upregulation of the mRNA expression of Abcc1, Abcc2, and Abcc4 at the BBB. This upregulation is regulated by the activation of nuclear factor E2-related factor (Nrf2) signaling. The Mrp isoforms belong to the ABCC group of proteins, and the enhanced functional expression of Mrp isoforms at BBB could induce the neural cell injury and death by reducing the concentrations of antioxidant glutathione (GSH) in the endothelial cell [107]. Another study demonstrated that the upregulation of Nrf2 by hyperbaric oxygen preconditioning (HBO-PC) might alleviate the hypoxia-ischemia brain damage (HIBD) [108]. Brain-derived neurotrophic factor (BDNF) is another critical molecule that promotes the growth and survival of nervous cells as well as the communication between neurons. In addition, it plays a major role in long-term memory and cognitive function. The circulating levels of BDNF in humans have been shown to increase dramatically during hypoxic stress, both in the perinatal period [109] and adulthood [110, 111] which, in turn, inhibits the ER stress activation as well as ROS production [112]. BDNF can regulate the ion channel activity and synaptic transmission in different regions of the brain by interacting with the TrkB receptor [113]. BDNF-TrkB signaling pathway has a precedent role in modulating the synaptic plasticity in the CNS [114]. During chronic hypoxia, the activation of BDNF-TrkB signaling pathway increases the voltage-dependent Ca^2+^ influx and catecholamine secretion in chromaffin cells [114]. Furthermore, CIHH pretreatment improved the ischemia-induced cognitive dysfunction by the activation of ERK1/2-CREB-BDNF signaling pathway [115].

As demonstrated above, BDNF not only plays a crucial role in the development of CNS but also regulates the plasticity of neurons during hypoxic stress.

STAT3 signaling pathway has also been shown to play a role in the hypoxic injury of neural cells. The hypoxic-ischemia damage may cause the phosphorylation of STAT3, and the activation of STAT3 signaling pathway might be involved in the apoptosis-mediated regulation of nerve cells [116], tissue loss, and gliosis following neonatal hypoxia-ischemia stress [117]. Hypoxia triggers cAMP/PKA signaling pathway in cortical astrocytes by releasing CRF, thereby leading to the activation of aquaporin-4 and cerebral edema [118]. In addition, the dysregulation of N-methyl-D-aspartate receptors (NMDARs)-Wnt-catenin signaling in the hippocampus may participate in the process of prenatal hypoxia that induces the spatial acquisition and retrieval deficits in adolescent offspring [119].

## 4. Summary and Conclusion

Neuronal plasticity is a critical property of the neural system regulating and coordinating mood and behavior. There are various intrinsic and extrinsic stimuli, which could affect the neuroplasticity in several aspects, such as the volume of nuclei, regulation of neuronal apoptosis and neurodegeneration, secretion of neurotransmitter, and any other forms of changes. Oxidative stress, inflammation, apoptosis, and excitotoxicity are the primary mechanisms underlying hypoxic-ischemic brain injury [10]. Various degrees of improvements were found in oxidative stress markers, cell density, the rate of neuronal apoptosis, and caspases in hypoxia-ischemia model of the rat after treatment with hyperbaric oxygen [120] and antioxidants. The goal was to explore the mechanism of hypoxic injury on the brain in order to focus on the long-term functional recovery in both injured and uninjured brain regions, and the neuronal plasticity might serve as the vital target.

## Figures and Tables

**Table 1:** Changes of neural plasticity induced by hypoxic-ischemic injury in various brain regions.

Brain region	Changes of neural plasticity	Mechanisms
Hippocampus	Synaptic plasticity	(1) Triggering of LTD via ROS and NOS (2) Impairment of LTP in presynaptic neurons (3) Impaired synaptic transmission and plasticity in dentate gyrus area of rats (4) Fine structural changes of synapses in CA1 region
	Volumetric changes	(1) Hippocampal atrophy and neurons apoptosis (2) White matter density decreased
	Apoptosis	(1) A severe loss of neurons induced by activation of MAPK signaling pathways (2) ER stress and oxidative stress
	Misfolded/unfolded protein	(1) Increased production of ROS

Prefrontal cortex	Synaptic plasticity	(1) Increased density of the GABAergic neurons impair the network activity within PFC (2) Increased neuronal death (3) Activation of T-type Ca^2+^ channels in the MD
	Activity in vmPFC and dlPFC	(1) Increasing the acetylcholine release by enhancing the activation of G protein
	Immunoreactive cells and cytokine changes	(1) Upregulated expression of molecules such as VEGF, Hif-1, and Glut1 (2) PV cells loss induced by the NOX2-derived oxidative stress
	Volumetric changes	(1) Increased neuronal death and ghost cells (2) Tissue atrophy

Amygdala	Synaptic plasticity	(1) Decreased expression of NPY (2) Increased expression of c-fos protein
	Volumetric changes	(1) Smaller gray matter volume in the amygdala and decreased volume of the left amygdala (2) Significant shrinkage of cell size and axonal degeneration of corticotropin-releasing factor-positive neurons

Signaling pathways	Upregulation of Abcc1, Abcc2, and Abcc4 mRNA expression at the BBB	(1) Regulated through Nrf2 signaling
	Activation of BDNF-TrkB signaling pathway	(1) The circulating levels of BDNF increased
	Enhancement of ERK1/2-CREB-BDNF signaling pathway	(1) The circulating levels of BDNF increased
	Activation of STAT3 signaling pathway	(1) Phosphorylation of STAT3
	Triggering cAMP/PKA signaling pathway	(1) Increased release of CRF
	Dysregulation of NMDARs-Wnt-catenin signaling	(1) Decreased expression of NMDARs
